# Digitalising behavioural data collection through cloud-based technology in veterinary science and beyond

**DOI:** 10.3389/fvets.2025.1600619

**Published:** 2025-06-19

**Authors:** Michelle Braghetti, Liat Vichman, Nareed Farhat, Daniel Simon Mills, Claudia Spadavecchia, Anna Zamansky, Annika Bremhorst

**Affiliations:** ^1^Clinical Anesthesiology, Department of Clinical Veterinary Science, Vetsuisse Faculty, University of Bern, Bern, Switzerland; ^2^Tech4Animals Lab, Information Systems Department, University of Haifa, Haifa, Israel; ^3^Animal Behaviour, Cognition and Welfare Group, Department of Life Sciences, University of Lincoln, Lincoln, United Kingdom

**Keywords:** digital data collection, mobile app for behavioural research, veterinary pain assessment, dog pain, dog pain behaviour, Dog Pain Database, mobile app for research, clinical data collection tool

## Abstract

Field data collection in veterinary and animal behaviour science often faces practical limitations, including time constraints, restricted resources, and difficulties integrating high-quality data capture into real-world clinical workflows. This paper highlights the need for flexible, efficient, and standardised digital solutions that facilitate the collection of multimodal behavioural data in real-world settings. We present a case example using PetsDataLab, a novel cloud-based, “no code” platform designed to enable researchers to create customized apps for efficient and standardised data collection tailored to the behavioural domain, facilitating capture of diverse data types, including video, images, and contextual metadata. We used the platform to develop an app supporting the creation of the Dog Pain Database, a novel comprehensive resource aimed at advancing research on behaviour-based pain indicators in dogs. Using the app, we created a large-scale, structured dataset of dogs with clinically diagnosed conditions expected to be associated with pain and discomfort, including demographic, medical, and pain-related information, alongside high-quality video recordings for future behavioural analyses. To evaluate the app’s usability and its potential for future broader deployment, 14 veterinary professionals tested the app and provided structured feedback via a questionnaire. Results indicated strong usability and clarity, although agreement with using the app in daily clinic life was lower among external testers, pointing to possible barriers to routine integration. This proof-of-concept case study demonstrates the potential of cloud-based platforms like PetsDataLab to bridge research and practice by enabling scalable, standardised, and clinically compatible behavioural data collection. While developed for veterinary pain research, the approach is broadly applicable across behavioural science and supports open science principles through structured, reusable, and interoperable data collection.

## 1 Introduction

Emerging technologies are advancing data collection methods and transforming research across various fields, including veterinary and animal behaviour science. These innovations have unlocked novel research directions and opportunities for collecting, analysing, and applying data in unprecedented ways. For example, social media platforms like YouTube have become recognised as rich repositories of raw data, offering access to diverse animal behaviours captured in naturalistic settings by users worldwide [e.g., (1–4)]. Researchers can leverage this wealth of online content to study animal behaviours that would otherwise be challenging or costly to observe in the field. Other web-based tools such as the Gorilla Experiment Builder^1^ enable researchers to design and execute experiments online with precision, addressing challenges like timing, sample diversity, and accessibility (5).

These advancements illustrate the growing importance of efficient and scalable methods for data collection across diverse scientific domains, particularly in contexts where traditional data collection methods often face (practical) limitations. This is particularly the case beyond controlled laboratory settings in real-world environments. While laboratory settings offer precision and consistency, allowing for more controlled observations of behaviours, physiological changes, and medical conditions, they often lack the diversity and complexity found in the field. This can potentially limit the generalisability of findings to broader populations and real-world settings [see, e.g., (6–8)]. In contrast, field environments, including veterinary clinics, provide invaluable opportunities to observe animals in naturalistic conditions. Expanding data collection into these settings enables researchers to capture more diverse and representative datasets, including those from animals experiencing real-world challenges like pain, stress, or disease. Such diversity is crucial for understanding complex phenomena, such as the relationship between behaviour and underlying medical conditions [as described, e.g., in (9, 10)]. However, collecting data in such dynamic environments typically presents significant logistical and technical challenges. These include the need for practical solutions to handle large volumes of data, specialised recording equipment, and the standardisation of collection protocols across diverse and often unpredictable settings. Addressing these challenges is essential to maximise the potential of clinical and field environments for research purposes.

In the case of veterinary science, expanding data collection as described not only broadens the scope of research but also opens opportunities to involve practicing veterinarians as active contributors to the research process. Veterinarians may be regarded as the “eyes and ears” of the field, interacting daily with a wide variety of cases and diverse animal populations in real-world settings. This unique position allows them to observe and document behaviours and medical conditions that might otherwise go unnoticed in controlled research environments. The exposure of veterinarians to such diversity provides unparalleled opportunities for data collection, which is crucial for advancing the veterinary field (11).

Initiatives like VetCompass, first initiated at the Royal Veterinary College, UK, and in the meantime also introduced in Australia [VetCompass Australia, (12)], demonstrate the potential of involving veterinary practitioners in systematic data collection. VetCompass aggregates anonymous clinical data from participating practices, enabling large-scale research into common disorders, risk factors, and disease patterns (12). By standardising terminologies and integrating data across practices using tools like the VeNom Codes, VetCompass demonstrates how practitioners can actively contribute to research by integrating data collection into their daily practices and thereby providing valuable insights when supported by structured and efficient data collection frameworks (12).

Involving veterinary professionals as active contributors to data collection can therefore significantly enrich animal datasets with naturalistic and context-specific observations. This integration also helps bridge the gap between clinical practice and scientific research, fostering a collaborative approach that benefits both evidence generation and practical care. However, leveraging this potential requires addressing the realities of busy clinical workflows. For practitioners to contribute meaningfully, data collection must be streamlined, efficient, and easy to integrate into their routine. This calls for user-friendly tools that enable seamless capture of behavioural observations—such as videos or images—without the need for specialised equipment or time-intensive procedures. Thus, the challenge lies in developing innovative, standardised, and scalable solutions that accommodate the unique demands of clinical settings while empowering practitioners to participate in research with minimal disruption to their primary responsibilities.

Emerging technology, like the use of mobile apps and web-based platforms, offer promising new opportunities for data collection in clinical environments and beyond. Mobile apps, in particular, enable flexible and efficient real-time capture of diverse data types-including video, images, and contextual information-often requiring only a smartphone. This makes them particularly well-suited for busy, dynamic, and time-constrained and clinical environments.

Recent years have seen a growing interest in digital technologies across veterinary and animal health contexts, including the integration of mobile platforms for clinical support, data collection, and client communication. Surveys from multiple countries show that veterinarians are increasingly using digital communication tools such as email, text messaging, and video consultations, and express openness toward mobile health applications and remote monitoring systems (13–15). Specific veterinary-focused apps have been developed, for instance, in India to support canine healthcare and client interaction (16), and m-health tools have been trialled to support treatment adherence in chronic conditions such as canine atopic dermatitis (15). However, these applications are typically designed for fixed clinical workflows or client-facing use cases and do not offer researchers the flexibility to design and control custom data collection protocols. To our knowledge, there is currently no platform in veterinary and animal behaviour research that enables researchers to independently create fully customised, multimedia-enabled data collection apps tailored specifically to behavioural or clinical research. While “no-code” solutions like Pathverse have emerged in human healthcare research (17), these are primarily focused on building apps for intervention delivery and behaviour change in human populations.

In response to this gap, we developed PetsDataLab, a cloud-based platform designed specifically to support the creation of tailored mobile apps for streamlined data collection in clinical and animal behavioural research. PetsDataLab supports the capture of diverse data types, including videos, images, audio, structured questionnaires, and metadata, all without the need for programming skills. Tailored apps can be designed through an intuitive, click-based interface in just a few steps.

In this paper, we describe the proof-of-concept use case of PetsDataLab: the development of a customised app to support the creation of the Dog Pain Database—a structured, multimodal dataset collected from dogs in clinical settings who were experiencing presumably painful conditions. The database integrates demographic, medical, pain-related, and high-quality video data and is intended to serve as a foundational resource for advancing canine pain assessment and supporting the future identification of behavioural pain indicators. We chose to pilot the platform in the domain of canine pain because pain is a particularly urgent and complex area in veterinary behavioural research, where objective, scalable tools are still lacking and improved assessment methods are critically needed to support both welfare and clinical decision-making. By showcasing this first use case, we also aim to demonstrate the potential of innovative digital solutions like PetsDataLab for overcoming practical challenges of data collection in veterinary science and beyond.

## 2 The PetsDataLab platform

PetsDataLab is a web-based platform designed to make the creation of custom-made mobile apps for multimedia data collection simple and effortless (2). The platform was originally developed to address two key scenarios involving animal-related data. The first scenario involves citizen science projects, in which members of the public actively contribute to scientific research by capturing photos or videos and providing relevant information (e.g. such as geolocation) at any time and location. This makes mobile devices a convenient tool for flexible and widespread real-world data collection. The second scenario focuses on collecting clinical data from real patients, where integrating data collection seamlessly into the daily workflows of a clinic is critical, especially if data is collected by the practitioners themselves, as they are occupied with clinical duties and cannot invest significant time in additional tasks. While these two scenarios have distinct requirements, PetsDataLab is designed to be flexible and can effectively support both, providing a scalable and adaptable solution for diverse research and data collection needs.

Based on these scenarios, the platform is intended for two types of users:

1. **Researchers** initiate the data collection process by creating a custom-made mobile app on the PetsDataLab platform. They define the type and format of the data to be recorded, including any uploaded visual material (images/videos), and have full access to the collected data for further analysis. Once the app is created, it can be shared with a designated group of data collectors who carry out the actual data collection.
2. **Collectors** use the custom-made data collection app to engage in data recording activities.

**Figure 1:**
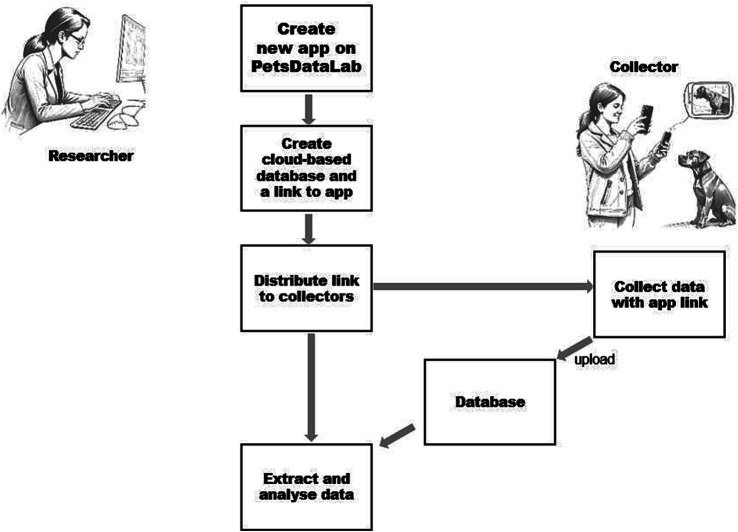
The typical workflow of creating and using the tailored data collection app created with the PetsDataLab platform.

Figure 1 presents a typical workflow of creating and using a data collection app with the PetsDataLab platform.

**Figure 2:**
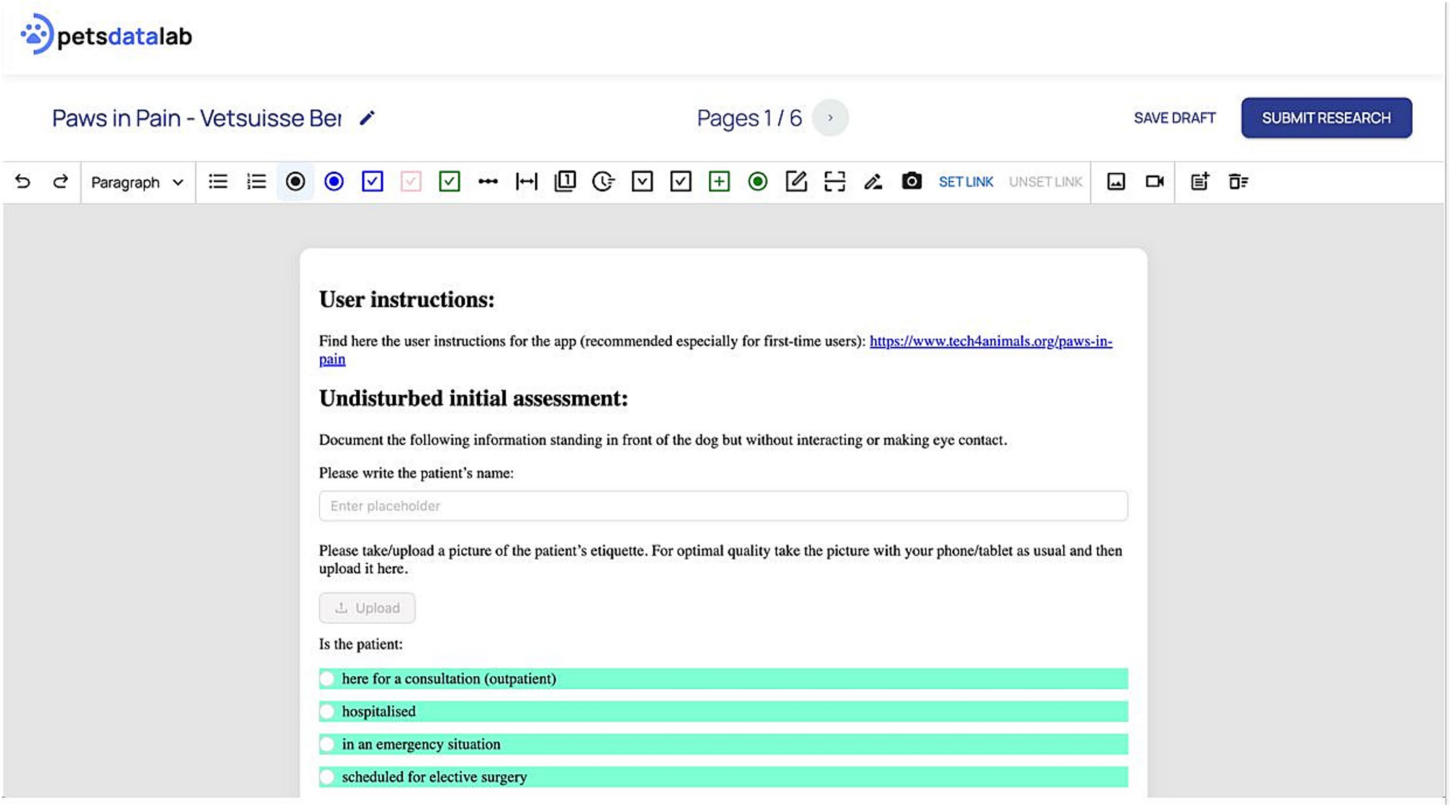
Screenshot of the PetsDataLab editor interface.

**Figure 3:**
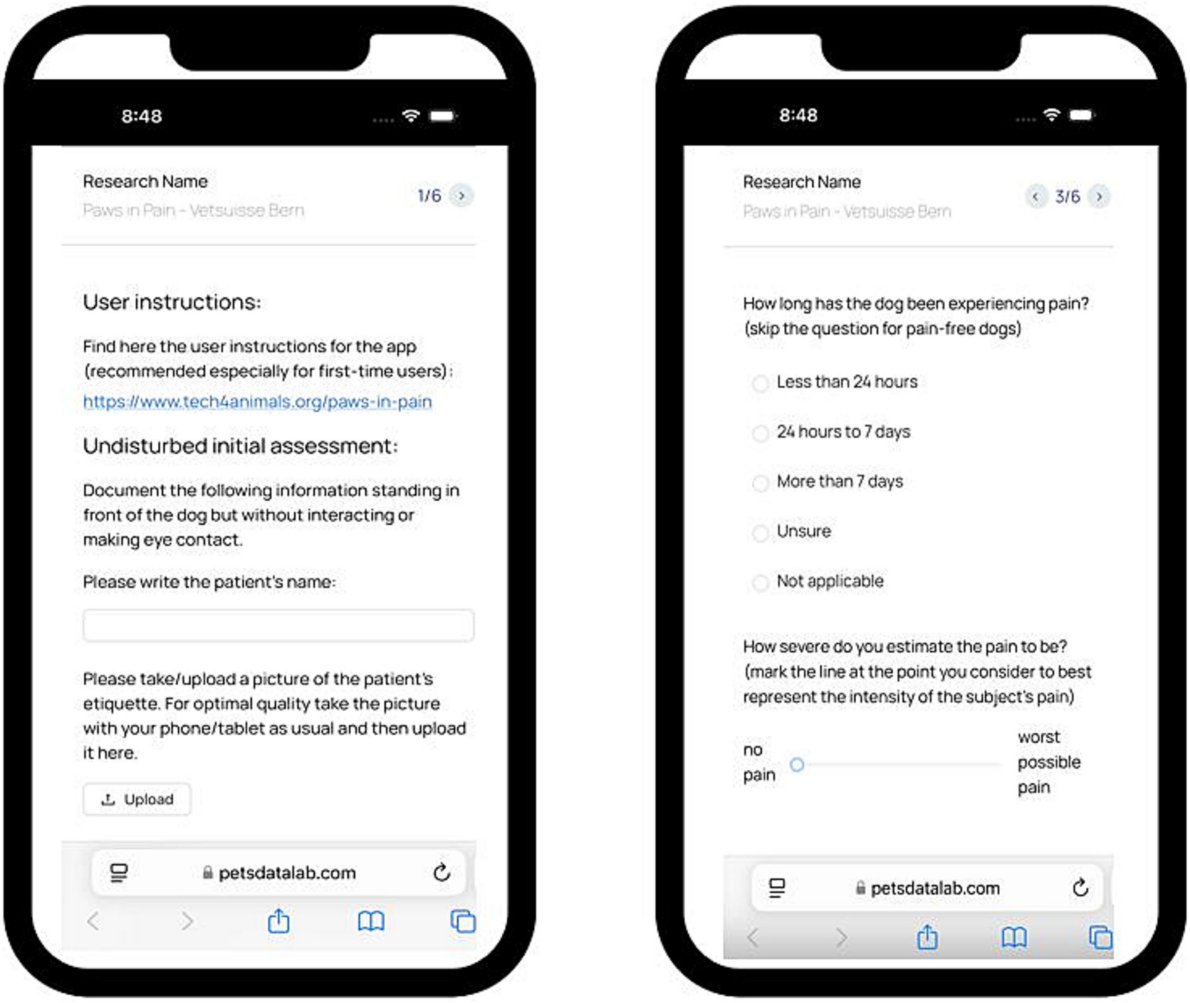
The collector’s view of the app in the mobile version. This figure shows a mock-up with a screenshot of the Dog Pain Database app.

The PetsDataLab platform^2^ consists of the following three main components:

1. Editor: a web-based interface for mobile app creation. This component is used by the researchers. It has functionality similar to Google Forms^3^, but offers extended capabilities specifically tailored to behavioural and clinical research. This includes a rich content editor that supports text input, multimedia uploads, and customizable questions and entry fields with the possibility of conditional logic, enabling the creation of both mobile and web-compatible apps. Figure 2 presents a screenshot of the editor interface.
2. App: the created custom mobile app for data collection. This component is used by the data collectors, who can access the app through a standard internet browser from a mobile device or computer desktop. No installation of the app is required as it is accessed via a link shared by the researcher. Figure 3 shows an example screen of the mobile app from the presented “Dog Pain Database” case study.
3. Data storage: the backend architecture of the platform is built on Firebase, providing robust data storage and real-time synchronisation. Amazon S3 is employed for media storage, allowing for efficient handling of image and video uploads, ensuring state of the art data security standards. This setup ensures that both structured (textual) and unstructured (multimedia) data can be managed in a unified environment. When a researcher creates a form in the app creation interface, a JSON object is generated representing the structure of the custom mobile and web app. This JSON object defines also the organisation of the database. Firebase is used to securely store text and metadata in a scalable way, while Amazon S3 efficiently handles images and video uploads provided by contributors.

## 3 First use case: using PetsDataLab to create an app for developing the Dog Pain Database

The first proof-of-concept use case of the PetsDataLab platform was to create a tailored app for data collection in the field of canine pain research to support the creation of the Dog Pain Database. This case study focuses on developing the infrastructure needed for large-scale data collection in a clinical environment, which will enable future studies to identify pain-specific behaviours and indicators in dogs.

### 3.1 Background

Pain is a critical condition that can severely impact on animal welfare and quality of life as if this protective mechanism persists over time, it can transform into a source of suffering (18, 19). In veterinary practice, understanding and effectively managing animal pain is a professional obligation and at the same time a considerable challenge (20). Since animals cannot verbally communicate pain and other internal states, alternative methods and measurable approaches are required to accurately infer what they are likely internally experiencing (21).

To support pain evaluation in clinical practice, a variety of pain assessment scales have been developed [see, e.g., (22–24) for reviews of pain assessment scales for cats and dogs]. While these scales are valuable tools, many rely on observer-based assessments of the veterinarian or the owner [e.g., (10, 22)]. Even though some scales have been evaluated for inter-observer reliability [e.g., (25), see for a discussion focusing on the Glasgow Composite Measure Pain Scale (19)], the fact that they rely on human judgement inherently introduces subjectivity. A range of factors—such as the observer’s prior experience and expertise or familiarity with the animal—can likely influence how behaviour is interpreted, not only affecting general behavioural assessments but likely extending to pain assessment as well [e. g. (19, 26–28)].

To enhance objectivity in pain assessment, there is a growing focus on using standardised methods for evaluating pain through quantifying associated behavioural expressions. Particularly facial behaviours have gained significant attention in this context as potential measurable indicators of pain (29–31). Advancements in AI-based pain detection have furthermore demonstrated the feasibility of using automated facial expression analysis in species such as cats (32) and horses (33). However, humans tend to focus disproportionately on facial cues when interpreting emotions—an example of attentional bias, where certain types of information draw more attention than others. This bias has also been observed in studies investigating how people perceive emotional expressions in dogs (34). Yet, as Leach et al. (35) pointed out, facial expressions may not always be the most accurate or efficient source of information when assessing pain in animals. Instead, triangulating information from different sources, including body posture and general behavioural tendencies (36), may offer valuable information for accurate interpretations and should not be overlooked when developing pain assessment tools.

Despite being one of the most common companion animals worldwide, dogs still lack equivalent behaviour-based tools for pain assessment. Developing such tools is particularly challenging in dogs due to their exceptional morphological diversity. This includes substantial variation in both body but also facial features, e.g. skull shape, facial musculature, ear types, and facial coat colour patterns (2, 37–39). A recent study in domestic cats highlights how morphological variation of facial features can interfere with the reliable detection of pain expressions: while facial changes associated with pain were distinguishable in domestic shorthair cats, these differences between painful and non-painful conditions were no longer detectable in a more morphologically diverse group. In some paedomorphic breeds, neutral facial configurations were even scored as more painful than actual pain expressions in other breeds—suggesting that human-driven selection may obscure or distort facial signalling (39).

Similar concerns may apply to dogs, where we are beginning to see first empirical evidence that morphology may influence both the production and perception of emotional expressions (2, 40, 45, 46). As a result, such variation complicates the development of standardised behaviour-based tools for dogs, as it likely influences how pain is expressed—an aspect that remains insufficiently studied and must therefore be carefully considered in tool development.

To develop reliable pain assessment tools for dogs, a comprehensive understanding of their behavioural expressions in pain states is essential. This includes investigating not only morphological variation but also the influence of factors such as pain type, pain location, intensity, sedation, underlying disease, medication, stress, and individual differences [e.g., (31)]. Many of these influences remain poorly understood, hindering the accurate detection of canine pain and the development of effective assessment tools. Addressing these challenges requires innovative approaches and the generation of robust, comprehensive datasets to advance pain research in dogs.

To support advancements in this field, we aimed to develop a novel resource—the Dog Pain Database—which contains systematically collected, large-scale data from dogs expected to experience diverse, diagnosed pain states in clinical settings. The database integrates high-quality video material for future in-depth behavioural analysis with a particular focus on facial expressions of pain using structured systems such as the Dog Facial Action Coding System (DogFACS; Waller et al. 2024) or facial landmarking (41), as well as demographic, medical, and pain-related information. To facilitate the complex process of gathering this data, we developed the Dog Pain Database app using the PetsDataLab platform. The app was specifically designed to address key challenges in large-scale data collection in variable, busy veterinary settings, while maintaining consistency across diverse clinical workflows.

The app integrates two traditional clinical pain assessment tools: the Visual Analogue Scale [VAS; (42)] and the Short Form of the Glasgow Composite Pain Scale [CMPS-SF; (43)]. The VAS allows for intuitive, observer-based rating of perceived pain intensity, while the CMPS-SF is a instrument that provides a structured evaluation of pain across multiple behavioural domains. Including both scales not only ensures clinical relevance but also enables future research to examine how behavioural expressions of pain relate to established clinical scoring methods. Such comparisons may help assess their validity and complementarity, ultimately supporting the development of more objective and standardised pain assessment tools.

While the app’s primary function was to facilitate data collection for this specific project, our long-term vision is to establish a comprehensive, scalable canine pain database with broader applications beyond this initial study. In the future, we aim to expand data collection efforts by a multi-centre approach involving several clinicians across diverse clinical environments. This would improve the diversity and generalisability of the dataset, allowing for a more nuanced understanding of pain behaviours across conditions and morphologies. As an added incentive for clinical users, the app includes real-time calculation of the CMPS-SF, providing immediate feedback that may support decision-making around analgesic treatment. By offering clinical utility and research potential, this feature may promote wider adoption of the app and facilitate sustained, large-scale data collection. We therefore also conducted a usability assessment to evaluate the app’s reception among veterinary professionals, assess its feasibility for broader implementation, and identify areas for improvement. The evaluation provided valuable insights into the app’s functionality, ease of use, and integration into clinical workflows.

In this case study, we present the development of the Dog Pain Database app, the contents of the Dog Pain Database, and the design and results of the usability assessment. Together, these components highlight how the app was tailored to facilitate effective and systematic data collection while addressing the practical challenges of large-scale research in clinical environments.

## 4 Methods

### 4.1 Ethical approval

Ethical approval for this study was granted by the University of Lincoln Ethics Committee under the approval reference UoL2024_18004. In compliance with the Swiss Animal Welfare Act (TSchG), this study did not require approval from the Committee for Animal Experimentation of the Canton of Bern (Switzerland) as the data were collected during routine diagnostic and therapeutic procedures. Dog owners provided consent for the use of their pets’ data for research purposes.

### 4.2 Subjects

The study included dogs presented to the Small Animal Veterinary Clinic of the Vetsuisse Faculty, University of Bern, with conditions expected to be associated with pain and discomfort.

To capture a broad range of pain experiences and clinical presentations, dogs were deliberately enrolled across four predefined patient categories: (1) those scheduled for surgery, (2) those admitted to the emergency department, (3) those seen for consultation appointments with potentially painful conditions, and (4) those hospitalised for ongoing care. The classification was based on the clinical context at the time of the first observation, although patients could shift categories during their stay—for example, if a consultation led to the decision for elective surgery.

As many patients as possible were enrolled, depending on the availability of the data collection team. Exclusion criteria focused on ensuring the feasibility of future facial expression analysis. Dogs were excluded if continuous muzzling was required due to aggressive behaviour or if surgical procedures considerably altered facial anatomy in a way that would compromise video-based analysis. We acknowledge that this may introduce selection bias by underrepresenting individuals who may express pain differently or more intensely. However, these exclusions were necessary to ensure the quality and interpretability of the visual data, which is central to the future aims of our work. Follow-up research should explore complementary strategies—such as alternative recording techniques or behavioural assessments not reliant on facial visibility—to include these important but methodologically challenging cases.

## 5 Data collection procedure

### 5.1 Data types collected

Several types of data were collected for the Dog Pain Database to enable addressing a range of different research questions in future studies. These data types included:

- Demographic data: breed, weight, age, sex, and neuter status.
- Medical and pain-related data: primary diagnosis, medical history, administered medications, relevant reports (e.g., anesthesia, radiology, orthopedic, or neurological examinations), patient category, and degree of sedation (in case of post-surgery observations). The data also included the presumed type and origin of pain, affected body part, and pain duration. In addition, we included the two traditional scales for pain assessment: (1) the VAS, a 100 mm line with endpoints labelled “No pain” (0) and “Worst possible pain” (100), where observers marked the perceived pain level to estimate pain intensity (42); and (2) the CMPS-SF (43) to assess acute pain in dogs, consisting of six questions to evaluate different aspects of the dog’s responses, including vocalisation, mobility, and interactions with people, as well as specific behaviours.
- Visual data:
  Images: photographs of patient clinic data sheets and protocols for later classification.
  Videos: to enable future behavioural analyses, each observation included recording the dog in two standardised conditions: a non-social context and a social context. In the non-social context, a one-minute video was taken of the dog’s face and body without any intended social interaction between the observer (the collector, Figure 1) and the dog, including avoidance of eye contact. In the social context, a video captured the dog’s first social interaction with the collector, following a standardised sequence that involved approaching the dog, speaking to her/him, and palpating various body areas—including the painful site (if possible) or, for pre-surgical patients, the planned operative area. Social videos were not recorded in cases where a secure approach was not possible, e.g., when dogs exhibited extreme distress, fear, or showed aggressive behaviour.

### 5.2 Data collection methods

- Location: data was collected in various sites within the veterinary clinic, depending on each patient’s category and condition. Locations included the Intensive Care Unit, the non-critical patient ward, anesthesia preparation and recovery rooms, consultation rooms, and the emergency room.
- Timing: data collection timing was tailored to each patient category:
  - Surgery cases: Before surgery and at predetermined intervals post-surgery (0–2 h, 2–4 h, 6–12 h, and >12 h after extubation), with daily follow-ups.
  - Emergency cases: before initial treatment and every two hours thereafter.
  - Consultation cases: before, during, and after treatment.
  - Hospitalised patients: once daily.

Data collection was subject to the availability of the data collection team, resulting in possible deviations from the predetermined optimal timing schedule. The data collection team was composed of two veterinarians (MB and CS) and a behavioural researcher (AB). The primary responsibility for data collection rested with one veterinarian (MB), who managed the majority of the data gathering efforts on a regular basis. The senior veterinarian (CS) and the behavioural researcher (AB) were not actively involved in daily data collection but provided continuous guidance, supervised the pilot phase, and supported methodological consistency throughout the study. This collaborative supervision helped ensure the accuracy and reliability of data for subsequent analysis.

### 5.3 Data sources

Data were collected using two primary sources:

- Dog Pain Database app: custom-developed for efficient and standardised collection of demographic, pain-related, and visual in-clinic data (see details below).
- Clinic’s patient management system: in line with our goal of making in-clinic data collection as efficient and time-saving as possible, the app was designed to capture only the information essential for behavioural analysis and pain assessment—particularly data that could not be retrieved from other sources. This approach helped reduce the time burden on clinicians and data collectors, increasing the feasibility of integration into routine clinical workflows. Additional demographic and medical information deemed potentially relevant for future analyses, but already available in structured form, was extracted from the clinic’s patient management system. This included details such as extended medical history, diagnostic reports, or treatment records that did not need to be manually entered again through the app. By limiting app-based data entry to non-redundant and behaviourally critical content, we aimed to optimise data quality while respecting the constraints of busy clinical settings.

### 5.4 App development and application

The development and implementation of the app, along with the organisation of the collected data into the Dog Pain Database, were structured into five project phases.

#### 5.4.1 Phase 1 (April – May 2024): app prototype development and piloting

The research project team, comprising researchers with expertise in veterinary anesthesiology, veterinary behaviour, behavioural biology and canine science, and software and AI development, collaborated to create a prototype of the tailored Dog Pain Database app using the PetsDataLab platform. The primary goal was to develop a user-friendly app specifically designed for data collection in pain-related research in dogs in a clinical setting. Throughout this phase, the team held regular meetings to refine the app’s interface, content, and functionality, ensuring it met the requirements of both researchers and veterinary professionals. The initial prototype included features for collecting detailed information on patient category, diagnosis, suspected pain type, main affected body part, and pain severity, including using the VAS and CMPS-SF scales. Additionally, the app was designed to capture data on the professional position of the data collector. The app further incorporated advanced functionalities to streamline data collection, including a conditional logical structure, where some questions only appeared depending on previous answers. It also supported multiple-choice and single-choice questions, as well as the collection of visual data, including videos and images.

During pilot testing, the team identified and resolved bugs, iteratively refining the app. Adjustments included expanding the list of pain types and refining questions to improve accuracy and comprehensiveness. By the end of this phase, a refined prototype had been developed, ready for more extensive testing and data collection. Importantly, we also made sure that the data collected in the previous iterations was not lost despite the changes introduced to its structure.

#### 5.4.2 Phase 2 (June – August 2024): data collection with refined app prototype

Data collection with the refined app prototype began in June 2024. Every observation started with a non-interactive part during which the first set of questions of the app was completed without any engagement with the patient, including avoiding eye contact. After completing the initial questions, first the non-social and then the social video was recorded. Efforts were made to minimise movement during video recording by using a tripod whenever possible. However, constraints such as cage construction and patient positioning sometimes made this impractical. In cases where the dog’s face was largely obscured by a cage door grid, data collection was rescheduled or conducted in a different room. Videos could be recorded directly through the app or uploaded separately into the app for optimal image quality. For patients capable of walking, the CMPS-SF assessment included observing the dog’s mobility on a leash. The data collection process concluded with taking a picture of the patient’s clinical protocol and submitting the data through the app. Throughout this phase, the team gathered feedback and held meetings to address challenges and further improve the data collection process.

#### 5.4.3 Phase 3 (September – November 2024): usability testing and final refinement

After the initial phase of data collection by the research team, the app was evaluated through a usability assessment in which a convenience sample of 14 veterinary professionals who had no prior exposure to the tool collected data with the app and filled in a usability assessment questionnaire. This group included 3 European College of Veterinary Anaesthesia and Analgesia (ECVAA) diplomates, 3 ECVAA residents, 4 veterinary interns, 2 veterinary students, and 2 veterinary nurses/technicians. Each tester was initially provided with detailed user instructions (see Supplementary material 1), which included navigation guidance and example videos demonstrating the social interaction component of the data collection. Testers were also encouraged to use the app at least twice to familiarise themselves with its features before completing a structured usability questionnaire designed by the project team (find the questionnaire in Supplementary material 2).

The questionnaire included both closed and open-ended questions and assessed several aspects of usability: whether the user instructions were read and perceived as clear, the app’s user-friendliness, clarity of the questions and answer options, overall impression of the app, time to complete the data collection, presence of any technical issues, and suggestions for improvement. Additionally, it evaluated the usefulness of the real-time CMPS-SF calculation and gathered feedback on the app’s potential for routine clinical use. Participants were asked how frequently they could imagine using the app in practice and whether they would recommend it to others. Responses were recorded using a 5-point Likert scale (1 = strongly disagree to 5 = strongly agree) or multiple-choice options where applicable. Each participant also indicated their professional background.

The majority of testers (*n* = 10, 71.4%) reported they read the user instructions before first testing the app and found them to be clear, giving a mean score of 4.7 out of 5 (median = 5; Table 1). Testers assessed the app as user-friendly, with a mean score of 4.3 (median score = 4.5) and rated the clarity of the questions and answers highly, with a mean score of 4.6 (median score = 5; Table 1). The time required to complete one observation, including video recordings, varied across participants. Half of the testers (50%) completed an observation in 10–15 min, while 42.9% required 5–10 min, and 7.1% needed 15–20 min.

**Table 1:** Summary of user feedback on the usability of the Dog Pain Database app, based on responses from 10 to 14 veterinary professionals (depending on question).

Question	*N* responses	Mean score	Median score	Strongly agree (score 5)	Agree (score 4)	Neutral (score 3)	Disagree (score 2)	Strongly disagree (score 1)
The instructions were clear	10	4.7	5	8 (80%)	1 (10%)	1 (10%)	0	0
The app is user friendly	14	4.3	4.5	7 (50%)	4 (28.6%)	3 (21.4%)	0	0
The questions and answers were written clearly	14	4.6	5	9 (64.3%)	5 (35.7%)	0	0	0
Visualizing the Glasgow Pain Score after submitting the application was useful.	14	4.3	4	6 (42.9%)	6 (42.9%)	2 (14.3%)	0	0
I could imagine collecting data with the app in the daily clinic life	14	3.5	3	4 (28.6%)	2 (14.3%)	5 (35.7%)	3 (21.4%)	0

Ratings of these questions were given on a 5-point Likert scale (1 = Strongly disagree, 5 = Strongly agree). Mean and median scores are provided for each question, along with the distribution of responses in percentages.

Feedback on specific app features highlighted both strengths and areas for improvement. The calculation of the CMPS-SF was well received (mean: 4.3, median: 4; Table 1). However, the feasibility of integrating the app into daily clinical workflows for data collection was rated lower (mean: 3.5, median: 3; Table 1), potentially reflecting practical constraints such as time limitations. When asked about the frequency of data collection in a clinical setting, responses were evenly distributed, suggesting variability in how often veterinarians felt they could realistically integrate the app into their workflow. Three testers (21.4%) indicated they could use the app multiple times per day, while another three testers (21.4%) reported they could use it multiple times per week. An additional three testers (21.4%) stated they could use it once per week, and three testers (21.4%) anticipated using it less than once per week. Only two testers (14.3%) stated they could use it once per day, and none indicated that they would never use the app.

Despite some challenges in routine use, most testers expressed a positive overall impression of the app. When asked whether they would recommend the app to someone else, 85.7% (*n* = 12) answered “yes,” while 14.3% (*n* = 2) answered “no,” indicating that while the app was overall relatively well-received, there are still areas for improvement.

It is possible that the perceived usefulness and frequency of app use would be higher if clinicians were collecting data for their own research purposes, as personal ownership and relevance of the data may enhance motivation. This distinction was not explicitly assessed in our usability study but represents an important consideration for future research and broader implementation.

Feedback from the testers helped in further refining the app and identifying areas for improvement to enhance potential future usability for a broader audience. These refinements aimed to position the app as a practical tool for large-scale data collection that extends beyond the research project team, enabling its application across diverse clinical settings. By involving external evaluators in this phase, we ensured that the app meets the practical requirements of veterinary professionals while maintaining its primary function as a robust research tool.

#### 5.4.4 Phase 4 (November – December 2024): phase 2 data collection

The final version of the app^4^ (Supplementary material 3) was deployed for a second data collection phase starting in November 2024. This phase represents the culmination of iterative development and user testing, ensuring that the app is well-optimised for capturing high-quality, consistent, and reliable data across different clinical settings and patient categories.

#### 5.4.5 Phase 5 (January 2025–March 2025): data extraction and organisation into the Dog Pain Database

Following data collection, all gathered information was systematically extracted, processed, and compiled into the Dog Pain Database to ensure structured and accessible data management for future research. This phase involved organising the dataset, validating entries, and preparing the data for future in-depth analysis.

PetsDataLab exports data to an Excel file, with each app version of the current study’s dataset stored in a separate sheet. The file includes all fields where users have provided input and for uploaded data, it contains the corresponding storage path in our Amazon Web Services (AWS) S3 system. Future developments aim to allow researchers to customise the titles of exported fields, which are currently autogenerated based on the text preceding the data collection input. Additionally, we plan to integrate user-configurable S3 storage, enabling researchers to store data directly on their own storage systems.

In this first use case study, data from 95 dogs were collected (for detailed information, see Supplementary material Data sheet 1), comprising 43 females (45.3%) and 52 males (54.7%). Among the females, 79.1% (*n* = 34) were neutered, while 20.9% (*n* = 9) were intact. Among the males, 65.4% (*n* = 33) were neutered and 34.6% (*n* = 19) were intact. Subject ages ranged from 11 to 182 months (mean = 86.7 months, median = 92.5 months).

The sample represented a diverse range of dog breeds, comprising 44 different breeds including crossbreeds. The most frequently represented were Crossbreeds (*n* = 12) and French Bulldogs (*n* = 12), followed by Labrador Retrievers (*n* = 8) and Yorkshire Terriers (*n* = 5). A detailed breakdown of all breeds is provided in the Supplementary Table 1 and Supplementary material.

Regarding patient categories, the 95 individuals were classified as follows: 52 (54.7%) underwent surgery, 21 (22.1%) were consultation cases, 20 (21.1%) were hospitalised, and two dogs (2.1%) were emergency cases (Supplementary material). Of these 95 individuals, a total of 308 observations were performed, resulting in 599 video recordings (308 non-social videos and 291 social videos, Supplementary material). In 17 observations (5.5%), the social context video could not be recorded and in 9 observations (2.9%), the social context video was captured, but palpation of the painful area was not performed due to factors such as aggression, excessive excitement, or logistical constraints.

## 6 Discussion

This proof-of-concept case study illustrates how a tailored mobile app developed using the PetsDataLab platform can enable systematic behavioural data collection in clinical veterinary research. Specifically, the Dog Pain Database app was designed to collect high-quality, multimodal data from dogs with various diagnosed pain conditions in real-world clinical settings. The resulting Dog Pain Database currently contains demographic, medical and pain-related and high-quality video recordings—providing a comprehensive resource to support future studies on pain-related behaviours in dogs. Our next planned step is to make the integration of multimodal data more seamless and convenient, using more sophisticated databases as well as implementing data quality checks.

By capturing data on a wide range of individuals and clinical contexts, the Dog Pain Database provides unique potential for identifying behavioural indicators of pain and examining how pain type, anatomical location, and individual morphology may influence behavioural expressions. The inclusion of high-resolution visual data further enables in-depth analyses using tools such as the Dog Facial Action Coding System [DogFACS; (40)] and facial landmarks (41), and opens the door for training AI-based systems for automated pain detection. These developments could substantially advance efforts to establish more objective, scalable, and standardised approaches to canine pain assessment—benefitting both animal welfare and clinical decision-making.

Automated pain detection technologies are beginning to emerge in veterinary and animal science, particularly for horses (33), sheep (44), and more recently, cats (32). These systems commonly use facial expression analysis based on validated grimace scales or geometric morphometrics. However, their development has relied on high-quality video data annotated under standardised conditions—highlighting the importance of curated datasets like the Dog Pain Database as a foundation for similar advancements in canine pain research.

The primary goal of the Dog Pain Database app was to facilitate efficient and standardised data collection. Usability testing among veterinary professionals confirmed its research-focused utility in this regard. Participants praised the app’s user-friendliness, clarity, and logical question flow. The real-time visualization of the Glasgow Pain Score was identified as a clinically valuable feature for most of the testers. However, the participants’ evaluations also raised concerns about the time required for data entry, especially in busy clinical workflows. Nonetheless, the average completion time—over 10 min per observation across all testers—may be viewed as a potential barrier for routine clinical use of the app in its current version.

These findings underscore a central tension in clinical behavioural research: the need for rich, high-quality data versus the practical constraints of real-world veterinary settings. To enhance the app’s long-term clinical utility and scalability, future iterations should aim to streamline workflows—for example, by incorporating features like voice input and further automated data fields. Additionally, offering features that provide direct clinical benefit may serve as incentives for broader adoption. For instance, enabling the usage of video data also for the practitioner’s patient records could support longitudinal tracking of lameness or pain-related behaviour over time, thereby aiding treatment monitoring.

Despite these challenges, the Dog Pain Database app represents a successful research tool that has already generated a valuable dataset. Expanding its use across multiple clinics and contributors would not only enrich the database but also provide deeper insights into the variability of pain behaviours, improve cross-context comparisons, and strengthen future tool development. The dataset collected offers a strong starting point for identifying pain-specific behaviours and refining behavioural assessment strategies, even before further data are added.

This case study also provided essential feedback that informed the evolution and continued development of the PetsDataLab platform itself. The iterative collaboration between researchers and developers revealed specific requirements for (veterinary) behavioural research that might not have been anticipated initially—such as more advanced question logic and support for external video uploads. These needs were systematically addressed during the developmental process, demonstrating the value of user-driven design in building adaptable digital tools for research.

Compared to generic form-building tools like Google Forms or Airtable—which offer general-purpose form and spreadsheet capabilities—existing platforms often lack key features necessary for behavioural science. This include integrated multimedia uploads, conditional logic, structured data validation, or real-time synchronisation. Such capabilities are essential when collecting rich, multimodal datasets in dynamic research environments, particularly when visual behavioural data such as videos or images must be aligned with contextual metadata.

We developed PetsDataLab as a more specialised platform designed with these features in mind to meet the methodological and practical demands of behavioural and clinical research. Its ability to combine intuitive app creation with flexible, standardised data structures makes it a powerful tool for managing complex datasets in both controlled and real-world settings.

However, our broader message is not limited to any one tool: new digital platforms—especially those that merge spreadsheet-like flexibility with database-level functionality—are rapidly transforming the possibilities for behavioural data collection. When thoughtfully applied, such technologies can dramatically improve standardisation, scalability, and integration of behavioural and contextual data—regardless of the specific tool used.

During the development and testing of the PetsDataLab-based app, we encountered technical challenges, including a bug in a feature intended to scan and automatically save extracted text from alphanumeric fields. While this component functioned correctly during local testing, it failed in production across several mobile devices, leading to minor data loss. This issue, which was discovered during comprehensive testing and subsequently fixed, highlighted the importance of robust quality control at every development stage. It also underscored the necessity of rigorous cross-platform and end-to-end (E2E) testing, particularly for tools intended for diverse clinical environments. In response, we implemented enhanced data verification processes to improve the reliability of critical features and overall platform stability, systematically testing the app for different scenarios, both by user (e.g., closing before completion of upload, uploading 0, 1 or more images, etc.) and by researcher (e.g., downloading all data, removing a sample, etc.).

Looking ahead, we plan to further expand the platform’s capabilities by integrating AI-driven features, such as automated animal detection in video frames and real-time feedback to improve data quality during recording. We are also exploring the use of conversational AI to assist researchers in app creation and customisation. These innovations aim to lower barriers to entry, streamline research workflows, and expand adoption of digital tools in both scientific and applied settings.

While this study focused on a veterinary science application, the underlying approach is broadly applicable. PetsDataLab—and similar tools—offer scalable, accessible solutions for any context requiring structured, multimedia-based behavioural data collection, including wildlife monitoring, shelter or on-farm assessments, and citizen science. By enabling researchers and practitioners to build customised tools without programming expertise, such platforms help bridge the gap between research and practice and foster more inclusive, interdisciplinary participation in data-driven behavioural science.

Moreover, by enabling structured, standardised, and well-documented data collection, these tools support the principles of open science. They facilitate greater transparency, reproducibility, and the potential for data sharing and reuse across institutions and projects—paving the way for more collaborative and cumulative progress in behavioural research.

As digital tools continue to reshape research workflows, cloud-based platforms like PetsDataLab introduced in this work illustrate the potential to redefine how behavioural data are captured, standardised, and analysed. The technological landscape is evolving rapidly; researchers must remain agile and critical in choosing tools that align with their goals—while keeping the focus on improving data quality, reproducibility, and accessibility in veterinary science and beyond.

## Data Availability

The original contributions presented in the study are included in the article/Supplementary material, further inquiries can be directed to the corresponding author.

## Footnotes

^1^ www.gorilla.sc

^2^When PetsDataLab is first written at the beginning of this section. Available at https://www.petsdatalab.com/

^3^ https://www.google.com/forms

^4^ https://PetsDataLab.com/research/BKRIMBOVItTCMjx3xhFK/
