# Supplementary material for: Digitalising behavioural data collection through cloud-based technology in veterinary science and beyond
Source: Front Vet Sci. 2025 Jun 19;12:1600619. doi: 10.3389/fvets.2025.1600619 (PMC12223317; doi:10.3389/fvets.2025.1600619)
Supplement: Supplementary file 1 [file Data_Sheet_1.pdf]

Supplementary Material

1 Supplementary information

SM 1: App user instructions

The following document contains the user instructions for the Dog Pain Database app, which were provided to all app testers before they participated in the usability questionnaire. These instructions guided users through the app's features, data entry process, and video recording requirements to ensure standardized data collection:

Dear App User,

Thank you for using the PetsDataLab app that allows multi-site and multi-user data collection in research. Our goal is to develop a dog pain database by collecting data from dogs in different pain states.

By using the app, you consent to the use of the uploaded data for our research. All data is anonymous. Upon submission, you will also receive a Glasgow Composite Pain Scale score for immediate evaluation of the dogs' pain state.

Data collection: Takes about 10 minutes → answer up to 16 questions + take pictures and videos.

How often? Depends on patient's category, ideally:

- EMERGENCY: Once before initial treatment, then every 2 hours.
- SURGERY: Once pre- and multiple times post-surgery (ideally 0-2h, 2-4h, 6-12h, >12h after extubation) and once daily after.
- CONSULTATION: During each consultation for that specific problem.
- HOSPITALISED: Once daily.

Instructions

E-Mail: Enter your email. If the email address has already been used, choose between "Fill New Research Form" or "Continue with existing filled forms" and then choose the corresponding trail. This allows you to change your answers after submitting them.

Here are some notes to help you navigate through the pages:

- For not painful patients undergoing surgery, select "No Pain" in these questions

Where do you think the dog has pain (main affected body part)?	What type and origin of pain do you suspect (multiple answers possible)?

- Video recording:
  - Use a tripod if possible to avoid too much movement.
  - **Video 1 (no interaction):** 1 minute, no interaction with the dog, focus on filming the face and when possible include the whole body.
  - **Video 2 (interaction):** Look at the dog, say the name, wait for 5 seconds. Approach the dog, start gently palpating the neck, front legs, thorax, abdomen, hind legs, and then the painful area. For elective surgery cases, also palpate the operative site before surgery, even if not painful.
    Example videos of the palpation procedure
    - Palpation of painful area: apply gentle pressure 5 cm around the site. Use the dog's reaction to answer the following question:

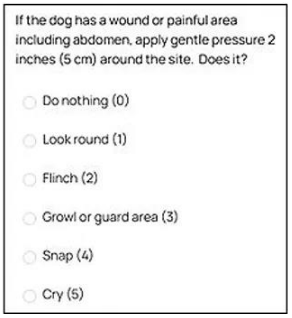

If the dog has a wound or painful area including abdomen, apply gentle pressure 2 inches (5 cm) around the site. Does it?

- Do nothing (0)
- Look round (1)
- Flinch (2)
- Growl or guard area (3)
- Snap (4)
- Cry (5)

    - Avoid learning over or stroking the dog from above.
    - Stop/omit filming if the dog is aggressive, very stressed, highly anxious or in severe pain when palpating.
- If the dog needs support to walk or can't be stressed (e.g., directly after airway surgeries), skip this part:

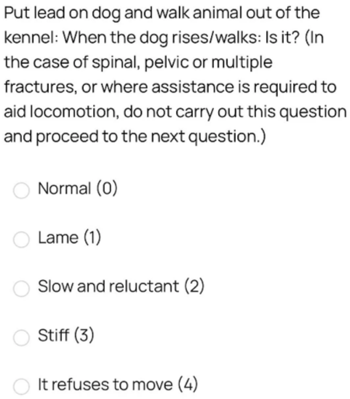

Put lead on dog and walk animal out of the kennel: When the dog rises/walks: Is it? (In the case of spinal, pelvic or multiple fractures, or where assistance is required to aid locomotion, do not carry out this question and proceed to the next question.)

- Normal (0)
- Lame (1)
- Slow and reluctant (2)
- Stiff (3)
- It refuses to move (4)

- If the dog is aggressive, and you skipped the palpation of the wound / painful area during the interaction, skip the question about it:

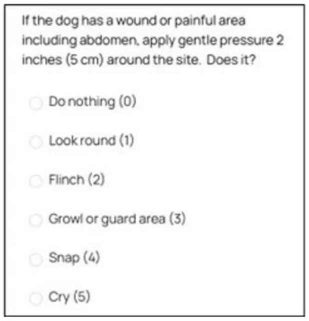

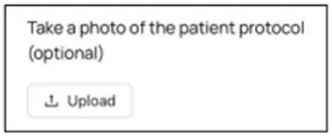

- Take a picture of the patient’s protocol (IPS Blatt).

SM2: App usability questionnaire

The following document contains the usability questionnaire that app testers were asked to complete after using the Dog Pain Database app:

User instructions

Have your read the user instructions before first using the app?

- Yes
- No

The instructions were clear.

- Scale 1 to 5 → 1 = Strongly disagree, 5 = Strongly agree.

What changes do you think could help improve the instructions?

- TEXT BOX

App usage

The app is user friendly.

- Scale 1 to 5 → 1 = Strongly disagree, 5 = Strongly agree.

The questions and answers were written clearly.

- Scale 1 to 5 → 1 = Strongly disagree, 5 = Strongly agree.

If some questions/answers were not written clearly: what was not clear?

- TEXT BOX

How long did it take you in total (including the filming part) to go through one application?

- 5-10 minutes
- 10-15 minutes
- 15-20 minutes
- >20 minutes

Were there any technical problems / bugs?

- Yes
- No

If there were any technical problems / bugs: what?

TEXT BOX

What changes do you think could help improve the app?

TEXT BOX

Glasgow Pain Score

Visualizing the Glasgow Pain Score after submitting the application was useful.

- Scale 1 to 5 → 1 = Strongly disagree, 5 = Strongly agree.

Future app use

Overall I could imagine collecting data with the app in the daily clinic life.

- Scale 1 to 5 → 1 = Strongly disagree, 5 = Strongly agree.

How often do you think you could collect data using the app in the clinic life?

- Multiple times per day
- Once per day
- Multiple times per week
- Once per week
- Less than once per week
- Never

Would you recommend the app to someone else?

- Yes
- No

Professional position

You are a:

- Veterinary nurse / technician
- Veterinary student
- Veterinary intern
- ECVAA resident
- ECVAA diplomate

Comments / suggestions

Do you have any comments / suggestions?

TEXT BOX

SM3: Final app version

The following document contains the final version of the Dog Pain Database app, including all questions and data points used for systematic data collection. Please also find the app here: https://petsdatalab.com/research/BKRIMBOVItTCMjx3xhFK/.

Page 1/6

User Instructions:

Find here the user instructions for the app (recommended especially for first-time users): https://www.tech4animals.org/paws-in-pain

Undisturbed initial assessment:

Document the following information standing in front of the dog without interacting or making eye contact.

Please write the patient’s name:

TEXT BOX

Please take/upload a picture of the patient’s etiquette. For optimal quality take the picture with your phone/tablet as usual and then upload it here.

UPLOAD BOX

Is the patient:

- here for a consultation (outpatient)

  What is the current situation?

  - First consultation (before treatment)
  - Follow-up (during treatment)
  - Follow-up (after treatment ended)

- hospitalised

  Data recording: Once per day.

- in an emergency situation

  What is the current situation?

  - Initial examination (before treatment)
  - Follow-up (after initial treatment treatment)

    Time since initial treatment:

    - 0-2 hrs
    - 2-4 hrs
    - 4-6 hrs
    - 6-12 hrs
- scheduled for elective surgery

  What is the current situation?

  - Pre-surgery
  - Post-surgery

    Time since the end of surgery:

    - < 2 hrs
    - 2-4 hrs
    - 4-6 hrs
    - >12 hrs

    Do you consider the dog t be fully recovered form anaesthesia?

    - No sedation (0)
    - Mild sedation (1)
    - Moderate sedation (2)
    - Profound sedation (3)

Page 2/6

If known what is the dog's diagnosis?

TEXT BOX

Where do you think the dog has pain (main affected body part)?

- Head
- Neck
- Back
- Pelvis

- Thorax
- Abdomen
- Front leg(s)
- Hind leg(s)
- Tail
- Paws
- Ears
- Eyes
- Unknown
- No pain
- Other

Please specify affected body part: TEXT BOX

What type and origin of pain do you suspect (multiple answers possible)?

- Acute
- Subacute
- Chronic
- Nociceptive
- Inflammatory
- Neuropathic
- Orthopaedic
- Visceral
- Somatic
- Unknown
- No pain
- Other

Please specify suspected pain type: TEXT BOX

Page 3/6

How long has the dog been experiencing pain? (skip the question for pain-free dog)

- Less than 24 hours
- 24 hours to 7 days
- More than 7 days
- Unsure
- Not applicable

How severe do you estimate the pain to be? (mark the line at the point you consider to best represent the intensity of the subject's pain)

No pain ________ Worst possible pain

Page 4/6

Take a 1 min video of the dog. Avoid disturbing and interacting with (incl. directly looking at) the dog. Focus on filming the face but try to film the whole animal.

UPLOAD BOX

Look at the dog in kennel/examination room: Is the dog?

- Quiet (0)
- Crying or whimpering (1)
- Groaning (2)
- Screaming (3)

Look at the dog in kennel/examination room: Is the dog?

- Ignoring any wound or painful area (0)
- Looking at wound or painful area (1)
- Licking wound or painful area (3)
- Chewing wound or painful area (4)

Film your first interaction with the dog: Have someone record you or set up your phone to capture the process (if unfeasible, skip filming). Approach and talk to the dog, gently palpate once the neck, thorax, abdomen, and extremities. When pre- or post-surgery, also palpate the operative site.

UPLOAD BOX

Page 5/6

Put lead on dog and walk animal out of kennel: When the dog rises/walks: Is it? (In the case of spinal. Pelvic or multiple fractures, or where assistance is required to aid locomotion, do not carry out this question and proceed to the next question.)

- Normal (0)
- Lame (1)
- Slow and reluctant (2)
- Stiff (3)
- It refuses to move (4)

If the dog has a wound or painful area including abdomen, apply gentle pressure 2 inches (5 cm) around the site. Does it?

Caveat: This question is part of the Glasgow Pain Scale – if it can’t be answered the calculated pain score is invalid and should not be used to decide whether additional analgesia is required or not.

- Do nothing (0)
- Lok round (1)
- Flinch (2)
- Growl or guard area (3)
- Snap (4)
- Cry (5)

Overall: Is the dog?

- Happy and content or happy and bouncy (0)
- Quiet (1)
- Indifferent or non-responsive to surroundings (2)
- Nervous or anxious or fearful (3)
- Depressed or non-responsive to stimulation (4)

Overall: Is the dog?

- Comfortable (0)
- Unsettled (1)
- Restless (2)
- Hunched or tense (3)
- Rigid (4)

Page 6/6

What is your professional position?

- Veterinarian
- Vet student
- TPA
- Animal caretaker
- Project team member

Take a photo of the patient protocol (optional)

UPLOAD BOX

Observations/comments

TEXT BOX

After submitting the application, the Glasgow Pain Score will appear.

Tab. SM1. Breed distribution in the Dog Pain Database

The dataset includes 95 dogs from a diverse range of breeds, reflecting the variability seen in clinical populations. The most frequently represented breeds include French Bulldogs (n=12), Labrador Retrievers (n=8), Yorkshire Terriers (n=5), and Dachshunds (n=4). Additionally, 12 mixed-breed dogs were included, making them the largest single category. In total, the dataset covers 44 different breeds (incl. crossbreeds), ensuring a broad representation of canine morphologies and clinical conditions.

Nr	Breed	N (counted individuals)
1	Crossbreed	12
2	French Bulldog	12
3	Labrador Retriever	8
4	Yorkshire Terrier	5

5	Dachshund	4
6	Golden Retriever	3
7	White Swiss Shepherd	3
8	Pomeranian	3
9	Australian Shepherd	2
10	Miniature Poodle	2
11	German Sheperd	2
12	Portuguese Water Dog	2
13	Miniature Schnauzer	2
14	American Bulldog	2
15	Bernese Mountain Dog	2
16	Malteser	2
17	Flatcoated Retriever	2
18	Norwich Terrier	1
19	Barzoi	1
20	Pyrenean Mountain Dog	1
21	Rottweiler	1
22	Shih Tzu	1
23	English Bulldog	1
24	Staffordshire Bull Terrier	1
25	Transylvanian Hound	1
26	Welsh Corgi Cardigan	1
27	West Highland White Terrier	1
28	Whippet	1
29	Australian Kelpie	1
30	Bearded Collie	1
31	Dogo Argentino	1
32	Lagotto Romagnolo	1
33	Bolonka Swetna	1
34	Jack Russell Terrier	1
35	American Staffordshire Terrier	1
36	Broholmer	1
37	Giant Schnauzer	1
38	Cairn Terrier	1
39	German Pinscher	1
40	Cavalier King Charles Spaniel	1
41	English Springer Spaniel	1
42	Great Dane	1
43	Saint Bernard	1
44	Newfoundlander	1
